# Acupuncture for smoking cessation: A systematic review and meta-analysis of 24 randomized controlled trials

**DOI:** 10.18332/tid/109195

**Published:** 2019-06-04

**Authors:** Jian-Hua Wang, Robbert van Haselen, Mei Wang, Guan-Lin Yang, Zhe Zhang, Maria E. Friedrich, Li-Qiong Wang, Ya-Qiang Zhou, Mei Yin, Cheng-Yu Xiao, A-Li Duan, Shu-Chun Liu, Bin Chen, Jian-Ping Liu

**Affiliations:** 1 Science and Technology Department, Liaoning University of Traditional Chinese Medicine, Shenyang, China; 2 Centre for Evidence-Based Chinese Medicine, Beijing University of Chinese Medicine, Beijing, China; 3 International Institute for Integrative Medicine, Kingston, United Kingdom; 4 School of Preclinical Medicine, Liaoning University of Traditional Chinese Medicine, Shenyang, China; 5 Affiliated Hospital, Liaoning University of Traditional Chinese Medicine, Shenyang, China; 6 Medical Library, Liaoning University of Traditional Chinese Medicine, Shenyang, China

**Keywords:** acupuncture, smoking cessation, randomized trials, systematic review, meta-analysis

## Abstract

**INTRODUCTION:**

We evaluate the effectiveness and safety of transdermal acupuncture by needles for smoking cessation.

**METHODS:**

A literature search for randomized controlled trials (RCTs) was performed in seven electronic databases from inception to February 2017. Meta-analysis was conducted using Revman 5.3.0 software. We used either a random effects model (REM) or a fixed effects model (FEM) for pooling data according to the result of a heterogeneity test (defined as significant if I^2^>75%). Trial sequential analysis (TSA) was applied by TSA 0.9.5.10 Beta software

**RESULTS:**

Twenty-four trials involving 3984 participants were included. The methodological quality was generally low. With regard to smoking abstinence, meta-analysis showed acupuncture was more effective compared to no intervention/waiting list for short-term (4 weeks) cessation (1 trial, RR=2.37, 95% 1.41, 3.97) and long-term (longer than 6 months) (2 trials, RR=2.66, 95% CI: 1.50, 4.70). Compared to acupuncture/auricular acupressure alone, acupuncture plus auricular acupressure showed more benefit for short-term cessation (3 trials, RR=1.52, 95% CI: 1.03, 2.25). Acupuncture plus auricular acupressure was more effective compared to sham acupuncture plus sham auricular acupressure for short-term cessation (3 trials, RR=2.50, 95% CI: 1.44, 4.33) and long-term (2 trials, RR=3.61, 95% CI: 1.37, 9.48). Acupuncture in combination with counseling, educational smoking cessation program or moxibustion had more benefit compared to acupuncture for short-term cessation (3 trials, RR=0.75, 95% 0.63, 0.91) and long-term (2 trials, RR=0.77, 95% CI: 0.56, 1.05), and TSA illustrated the cumulative Z-curve of this comparison for long-term across the traditional boundary of 5% significance and monitoring boundaries. No serious adverse events occurred

**CONCLUSIONS:**

Acupuncture combined with counseling, educational smoking cessation program or moxibustion was more effective than acupuncture as monotherapy with regard to long-term smoking cessation. Further, high quality trials are needed to confirm the result.

## INTRODUCTION

Tobacco use is one of the biggest public health threats worldwide and it is the leading cause of preventable death, disease and impoverishment^1^. The World Health Organization (WHO) reports that tobacco use causes the death of about 7 million people per year worldwide. More than 6 million of these deaths are associated directly with the use of tobacco while around 0.9 million occur in non-smokers who are exposed to secondhand smoke^1^. Although there is still insufficient evidence, it is possible that exposure to thirdhand smoke (defined as the residual contamination from cigarette smoke after a cigarette is extinguished^2^) also has detrimental effects on human health^3-5^. Tobacco use is one of the main risk factors for a number of chronic diseases, including cancer, lung diseases, and cardiovascular diseases^6^. More than 80% of 1 billion smokers live in low- and middle-income countries, where the burden of tobacco-related illness and death is heaviest^1^.

Quitting smoking at any time or stage is beneficial to a person’s health as well as to society^7^. The Policies on National Basic Public Health Services in 2017 set by National Health and Family Planning Commission of the People’s Republic of China clearly stated that reducing the prevalence of tobacco use among people aged 15 years and older was the key of the project. Although there are many policies^8-13^ and methods^14-25^ related to smoking cessation at present, it is difficult for smokers to quit mainly because they are dependent on the highly addictive nicotine in tobacco^26^, besides psychological, sociological, and other factors.

Currently recommended intervention methods of smoking cessation include medications, nicotine replacement therapy (NRT), hypnosis, education, behavioral intervention, etc. Their applications are limited because of limited efficacy, side effects, or high price. Traditional Chinese Medicine (TCM) therapy, including auricular acupressure, body acupuncture, auricular acupuncture, herbal formulas, qigong, etc., is widely used to aid smoking cessation. Acupuncture plays therapeutic role by regulating the internal organs and meridians^27^ and has the advantages of a simple operation, few side effects and low expenditure^28,29^. Acupuncture has played an important part in the field of smoking cessation^30-33^ by suppressing the addiction and eliminating withdrawal symptoms^34^. Several systemic reviews have been conducted to evaluate the efficacy of acupoint stimulation for smoking cessation. However, their conclusions are not uniform^35-41^ and they need to be updated. The latest review was published in 2015 with a retrieval cutoff-point of December 2013 and also the Chinese literature search is not sufficiently represented in these systemic reviews. Besides, there is a lack of systematic review focusing on the efficacy of transdermal acupuncture for smoking cessation.

This review aimed to evaluate the evidence of transdermal acupuncture by needles for smoking cessation on the abstinence rate and on all the secondary outcomes reported.

## METHODS

This study was a systematic review and meta-analysis of previously published controlled trials, conducted and reported in adherence with the Preferred Reporting Items for Systematic Reviews and Meta-Analyses (PRISMA)^42^ guideline.

### Search strategy

Two library staff (SCL and BC) developed a search strategy for retrieving controlled trials on the efficacy of acupuncture for smoking cessation. A comprehensive literature search was conducted in two English and five Chinese databases: PubMed, the Cochrane library, China National Knowledge Infrastructure (CNKI), Chinese Biomedical Database (SinoMed), Wanfang, VIP Database and TCM online. These databases were searched from their inceptions to February 2017. In order to capture all relevant articles, search terms were intentionally broad (Appendix 1).

### Inclusion/exclusion criteria

#### Types of studies

RCTs, quasi-RCTs, and controlled trials that referred to the word ‘random’ in the method section, comparing acupuncture with either no intervention/ waiting list, sham acupuncture or other interventions, were eligible for inclusion. Studies that compared acupuncture in conjunction with another intervention to other interventions were also considered for inclusion in this review. We excluded cohort studies, case series, case reports, reviews, editorials, letters, commentaries, and animal studies. Studies for which we could not identify a full text or that did not report the minimum information required were excluded.

#### Types of participants

Tobacco smokers who wished to stop smoking.

#### Types of interventions and comparisons

The interventions included transdermal acupuncture by needles with or without additional application of electrostimulation or laser therapy. The acupuncture points used could be on the body, ear, face or head, delivered as a monotherapy or in combination with other interventions (NRT, acupressure, etc.). These interventions could be compared to no intervention/ waiting list, placebo, or other interventions. Studies on acupuncture-related therapies without needles inserted into acupoints were excluded.

#### Types of outcome measures

Outcomes included measures of efficacy (primary and secondary outcomes, as given below) and safety (adverse events) of acupuncture for smoking cessation. The studies were divided into three categories based on the follow-up duration: short-term for assessment up to 4 weeks, mid-term for an assessment duration up to 6 months, and long-term if outcome was assessed for longer than 6 months. Participants dropped out or lost to follow-up mainly due to moving or changing contact information were not counted in efficacy analysis.

##### Primary outcomes

The primary outcome was short-, mid-, and long-term abstinence from smoking. We preferred continuous abstinence (defined as abstinence between quit day or predetermined grace period and a follow-up time)^43^ to point abstinence (defined as prevalence of abstinence during a time window immediately preceding the follow-up)^43^. We also preferred biochemically verified abstinence (e.g. breath carbon monoxide, urinary cotinine, or both) to self-reported abstinence. If multiple assessments were available within one of the three follow up duration periods, the data obtained at the longest follow-up point was preferred.

##### Secondary outcomes

The secondary outcomes were nicotine withdrawal symptoms (NWS), the Fagerström test for nicotine dependence (FTND), the Beck Depression Inventory (BDI) and the Beck Anxiety Inventory (BAI), exhaled carbon monoxide (CO) level, cotinine content (in serum, urine or saliva), daily cigarette consumption, craving for cigarette, Heaviness of Smoking Index (HSI), brief questionnaire of smoking urges (QSU-Brief), and adverse events if reported.

### Study selection and data extraction

Two review authors (JHW and ALD) independently selected studies based on the inclusion/exclusion criteria. Data were extracted by two authors (JHW and ALD), independently, from the included studies using a self-developed electronic data extraction form. Data extracted included study location, sample size, participant characteristics, intervention details, outcome measures, and information on risk of bias. If a study had multiple intervention arms, data could be reused in different subgroup comparisons. Any disagreements were resolved by discussion with JPL.

### Assessment of risk of bias in included studies

The methodological quality of the included trials was independently analyzed in detail by two authors in order to assess the strength of the evidence. Any disagreements were resolved by discussion with JPL. The Cochrane Risk of Bias tool for systematic reviews of interventions was used to evaluate the methodological quality of individual studies (Cochrane Handbook for Systematic Reviews of Interventions Version 5.1.0 [updated March 2011] http://handbook.cochrane.org.). The risk of bias in each trial was rated as low, high, or unclear, based on the following items: random sequence generation (selection bias), allocation concealment (selection bias), blinding of participants and personnel (performance bias), blinding of outcome assessors (detection bias), incomplete outcome data (attrition bias), selective reporting (reporting bias) and other bias.

### Data analysis

The measures of effectiveness were estimated by mean difference (MD) with 95% confidence interval (CI) for continuous data. We calculated the risk ratio (RR) with 95% CI for dichotomous data. Heterogeneity, which was quantitatively assessed using the Cochran Q and I^2^ statistics to evaluate whether the pooled studies represent a homogeneous distribution of effect sizes, with I^2^≥75% considered as significant. RevMan 5.3.0 software (Copenhagen: The Nordic Cochrane Centre, the Cochrane Collaboration, 2014) was used for the data analyses. We performed random-effects meta-analysis for pooling data with significant heterogeneity and fixed-effects meta-analysis for pooling data without heterogeneity. We further performed subgroup analysis according to the type of experimental and control interventions if the data were available. Results were considered as statistically significant for p<0.05. We assessed publication bias using funnel plots in accordance with the Cochrane Handbook. TSA were performed by TSA 0.9.5.10 Beta software (Copenhagen: The Copenhagen Trial Unit, Center for Clinical Intervention Research, 2017) to calculate the required sample size and to detect the robustness of the result.

### Ethics approval and consent to participate

The present study did not require approval of an ethics committee or consent to participate, since the extracted data were obtained from publicly available publications.

## RESULTS

### Description of studies

**Figure 1:**
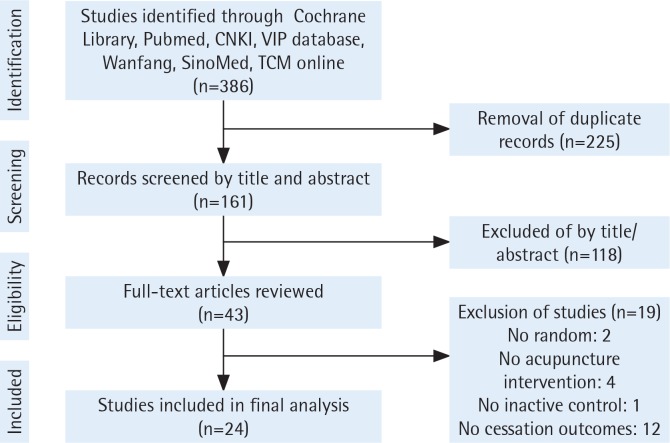
Literature screening flow

The literature screening flow is shown in Figure 1. Twenty-four studies that qualified for inclusion were identified in the review, yielding 27 comparisons in the intervention-level meta-analysis. Of the 24 included trials, 11 were published in Chinese^44-54^, and the remainder in English^55-67^. Ten were conducted in mainland China^44,46-54^, 3 in France^58-60^, while Taiwan^66,67^, USA^56,63^, UK^64,65^, and Korea^57,62^ each carried out 2 trials. Italy^55^, Norway^61^ and Malta^54^ each conducted 1 trial. Research in this area has been conducted over a long period since 1982^63^. Three trials were published in the 1980s and 1990s each, seven in the 2000s and eleven in the 2010s. Nineteen studies were published in journals and five as dissertations.

A total of 3984 participants were included in this review, and the characteristics of 24 included trials are summarized in Table 1.

**Table 1:** The characteristics of 24 included trials

*Study*	*N*	*Study arm*	*Description study arm*	*Male/ Female*	*Age (years) Mean±SD / Median (range)*	*Smoking history (years) Mean±SD / Median (range)*	*N Cigarettes daily Mean±SD / Median (range)*
Baccetti et al.^55^ (2015)	477	I	True TCM (included pharmacupuncture, plum-blossom needling and auriculotherapy)	59/103	50.0±10.4 / 50(28–85)	32.0±10.1 / 32(11–65)	21.7±9.1 / 20(10–60)
		C-1	True TCM and counselling	57/102	51.4±10.3 / 51(26–75)	33.2±10.8 / 33(10–58)	23.3±9.4 / 20(10–60)
		C-2	Sham TCM and counselling	56/100	47.8±9.1 / 48(28–71)	30.8±9.2 / 31(10–55)	23.3±10.4 / 20(10–80)
Bier et al.^56^ (2002)	141	I	True acupuncture and education	Total 45	NA/46.4(26–81)	NA/15.7(5–30)	NA/27.2(6.4–80)
		C-1	Sham acupuncture and education	Total 58			
		C-2	True acupuncture only	Total 38			
Chae et al.^57^ (2010)	29	I	Acupuncture	15/0	27.9±2.7/NA	NA	NA
		C	Sham acupuncture	14/0			
ClavelChapelon et al.^59^ (1997)	996	I	Nicotine gum and acupuncture	Total 548	NA/34(NA)	18±9/NA	NA
		C	Placebo	Total 448			
Clavel^58^ (1985)	651	I	Acupuncture	Total 224	NA	NA	NA
		C-1	Nicotine gum	Total 205			
		C-2	No intervention	Total 222			
Cottraux et al.^60^ (1983)	558	I	Acupuncture	108/32	NA/NA (30–40)	18.6±0.7/NA	31.3±1.0/NA
		C-1	Behaviour therapy	105/33		18.0±0.7/NA	31.5±0.9/NA
		C-2	Placebo medication	105/35		17.7±0.7/NA	31.6±1.1/NA
		C-3	Waiting-list	118/22		18.5±0.7/NA	30.9±1.1/NA
He et al.^61^ (2001)	46	I	Acupuncture	8/18	37.0±10.0/NA	21.0±9.0/NA	22.0±5.0/NA
		C	Sham acupuncture	10/10			
Hyun et al.^62^ (2010)	80	I	Acupuncture	36/2	NA/40.0(34.0–46.0)	NA/20.0(15.0–28.0)	NA/20.0(20.0–30.0)
		C	Sham acupuncture	39/3	NA/42.0(34.0–49.0)	NA/22.5(15.0–30.0)	NA/20.0(20.0–30.0)
Steiner et al.^63^ (1982)	32	I	Acupuncture	NA	NA	NA	NA
		C	Sham acupuncture				
Waite & Clough^64^ (1998)	78	I	Electroacupuncture using two needles at an active site plus self-retained ear seeds	22/18	NA/40.0(24.0–67.0)	NA/26.0 (5.0–53.0)	NA/NA(16.0–25.0)
		C	Electroacupuncture using two needles at a placebo site plus self-retained ear seeds	21/17	NA/45.0(23.0–69.0)	NA/28.0 (4.0–53.0)	
White et al.^65^ (1998)	76	I	Electroacupuncture	17/21	40.8±10.9/NA	22.6±9.5/NA	Most<30.0
		C	Sham electroacupuncture	20/18	42.5±13.9/NA	23.8±13.0/NA	
Wu et al.^66^ (2007)	118	I	Auricular acupuncture	48/11	54.3±16.9/NA	33.8±18.3/NA	16.7±10.6/NA
		C	Sham acupuncture	52/7	53.0±16.9/NA	32.8±17.4/NA	20.7±13.1/NA
Yeh et al.^67^ (2009)	59	I	Auricular electron acupuncture plus auricular acupressure	Total 30	28.0±7.8/NA	11.0±6.9/NA	14.8±6.4/NA
		C	Sham auricular electron acupuncture plus auricular acupressure	Total 29	27.0±7.6/NA	11.0±7.1/NA	16.6±5.7/NA
Bai & Ren^53^ (2001)	80	I	Acupuncture	40/0	NA/NA(19–23)	NA/NA(1–5)	NA/NA(5–30)
		C	No intervention	40/0			
Han^52^ (2006)	42	I	Acupuncture plus auricular acupressure	Total 22	M: NA/NA(23–70)	>10	NA/NA(20–40)
		C	Auricular acupressure	Total 20	F: NA/NA(19–72)		
Huang^51^ (2012)	60	I	Acupuncture plus auricular acupressure	27/3	33.9±8.6/NA	11.9±7.1/NA	22.7±5.3/NA
		C	Sham acupuncture plus auricular acupressure	25/5	31.3±7.3/NA	9.7±5.5/NA	21.0±4.9/NA
Liang^50^ (2013)	60	I	Acupuncture	Total 30	35.5±13.5/NA(20–58)	NA	NA
		C	NRT nicotine replacement therapy	Total 30	36.0±14.0/NA(26–50)		
Liu et al.^49^ (2015)	48	I	Acupuncture plus auricular acupressure	20/4	42±8/NA(20–70)	25±7/NA(2–46)	15±7/NA (15–50)
		C	Auricular acupressure	18/6	40±6/NA(18–65)	24±5/NA(2–44)	13±5 /NA(15–46)
Peng^48^ (2015)	60	I	Acupuncture plus auricular acupressure	27/3	44.1±12.2/NA	23.0±11.0/NA	17.1±6.2/NA
		C	Nicotine paste (NRT)	26/4	46.0±12.0/NA	24.8±11.6/NA	16.8±6.3/NA
Wang & Song^47^ (2013)	60	I	Acupuncture plus Moxibustion	15/15	NA/49 (40–55)	>20	NA /NA(15–20)
		C	Acupuncture	16/14	NA/46 (39–55)		
Wu^54^ (2015)	62	I	Acupuncture plus auricular acupressure	27/3	47.8±12.9/NA	27.1±10.7/NA	most 11–20
		C	Nicotine paste (NRT)	30/2	47.6±12.7/NA	27.0±13.6/NA	
Zhang et al.^46^ (2004)	60	I	Wrist-ankle acupuncture	30/0	25.7±4.4/NA	8.9±4.3/NA	25.8±7.2/NA
		C	Acupuncture	30/0	25.4±3.7/NA	8.7±3.5/NA	24.7±7.2/NA
Zhou & Wang^45^ (2010)	60	I	Electron acupuncture plus auricular acupressure	13/17	40.7±10.12/NA (20–60)	12.9±5.22/NA(1–30)	NA
		C	Electron acupuncture	13/17	40.3±9.78/NA(20–60)	13.0±4.67/NA(1–30)	
Li^44^ (2016)	50	I	Acupuncture	25/0	NA	23.96±12.03/NA	18.33±7.16/NA
		C	Nicotine paste (NRT)	25/0		21.61±11.47/NA	18.74±7.07/NA

N: number, I: intervention, C: control, M: male, F: female, NA: not applicable, SD: standard deviation.

### Experimental interventions

All studies in the review used a traditional approach to acupuncture with needles inserted into acupoints identified as specific for smoking cessation^54^ (Fei Shu – BL13, Shen Men – HT7, etc.). The form of acupuncture, however, varied greatly. Four studies^44,50,57,62^ used body acupuncture, two studies^63,66^ employed auricular acupuncture, one study^53^ adopted head acupuncture, one study^46^ employed wrist-ankle acupuncture alone, two studies^56,61^ used body and ear acupuncture, one study^58^ adopted head and eye acupuncture, and one study^60^ used body, ear and head acupuncture. Five studies^48,49,51,52,54^ combined body acupuncture and auricular acupressure, one study^47^ applied body acupuncture plus moxibustion, one study^59^ used body acupuncture plus nicotine gum. Additional current devices (such as electricity, seeds) were adopted to enhance the stimulation of acupuncture. One study^65^ investigated body electroacupuncture, two studies applied body electroacupuncture plus auricular acupressure with seeds^64^ or magnetic beads^45^, and one study^67^ used auricular electroacupuncture plus auricular acupressure with embedding seeds. One study^55^ investigated TCM intervention consisting of pharmacopuncture, plum-blossom needling and auriculotherapy.

### Control interventions

Nine studies^51,57,61-67^ used non-acupuncture points or points considered to have no specific effect on smoking cessation as a sham control intervention. NRT of known effect was adopted as a control intervention in four studies^44,48,50,54^. One study had a no-treatment control^53^. Five studies^45-47,49,52^ compared different acupuncture therapies with each other.

Nineteen studies had two arms. Five studies had more than one control group and therefore qualified for more than one comparison. One study had three arms: acupuncture, nicotine gum and no intervention^58^. The data of acupuncture compared with nicotine gum or no intervention were analyzed in separate pooled groups. Another study with three arms compared true TCM with true TCM combined with counselling or sham TCM combined with counseling, which were handled as two comparisons in different pooled analyses^55^. Two studies had four arms^59,60^ and one had three arms^56^, but only one comparison with data that could be pooled analyzed in every study was adopted in the meta-analysis.

### Risk of bias assessment

**Figure 2:**
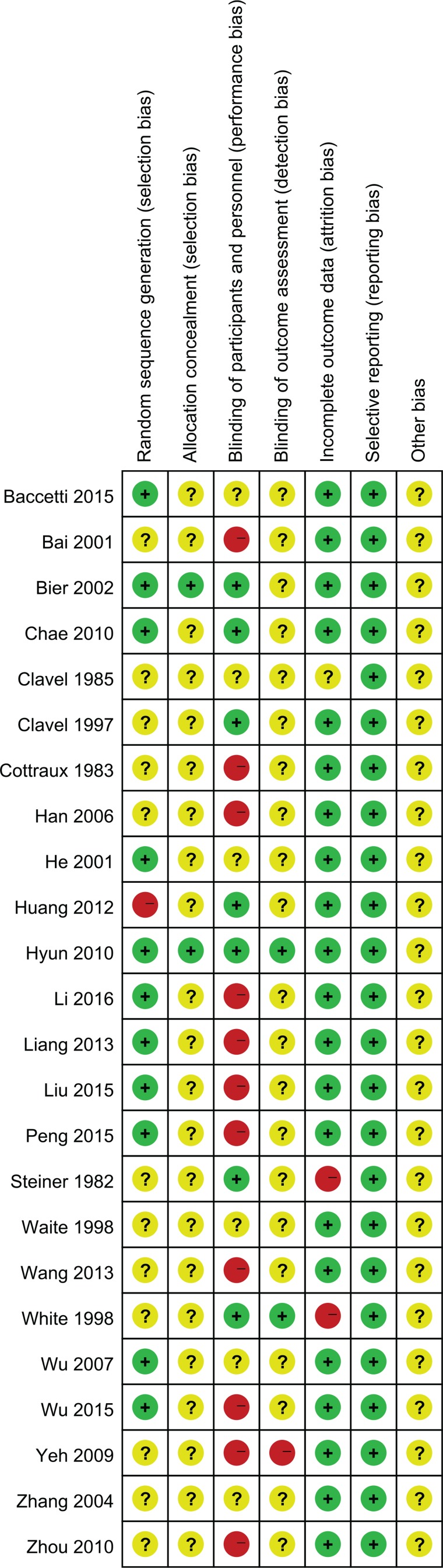
Risk of bias summary

**Figure 3:**
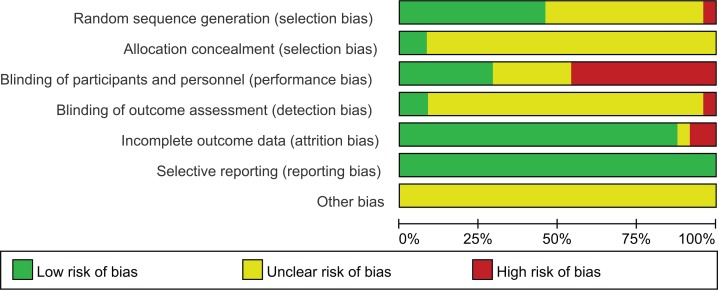
Risk of bias graph

The overall quality of evidence was low for all outcomes. The risk of bias was evaluated as unclear in most studies, except for selective reporting, because of lack of detail in the reports. The assessments of risk of bias for included studies are given in Figures 2 and 3. Eleven studies (11/24) described the random sequence generation and were judged as low risk of bias. Only two trials (2/24) reported allocation concealment while detailed information was deficient in the other 22 trials. There were 7 trials (7/24) that described blinding of participants and personnel clearly being judged to be ‘Low’ in this domain, and 11 trials (11/24) were judged to be ‘High’ because of the huge differences of interventions between groups. The majority of trials (21/24) provided no details related to blinding of outcome assessment, except for two (2/24) judged to be ‘Low’ and one (1/24) judged to be ‘High’. We considered 21 trials (21/24) as low risk of bias for incomplete outcome data according to the reports of the drop-outs or intention-to-treat analysis. Selective reporting was judged by the consistence between the outcome measures described in the method section and the actual outcomes in the result section, due to the deficiency of protocol information of all trials. All trials were assessed as low risk of bias for selective reporting.

### Effects of interventions

Based on the type of experimental and control interventions, we conducted meta-analyses for 9 different comparisons. Every outcome was reported in three subgroups: short-term, mid-term and long-term follow-up, where data were available (Table 2).

**Table 2:** Effect estimates of acupuncture for smoking cessation

*Outcomes Cessation rate = Primary outcome*	*Follow-up duration*	*Intervention group (N)*	*Control group (N)*	*Study (N)*	*RR/MD ( 95% CI)*
**1. Acupuncture versus no intervention/waiting list**					
Abstinence rate	**short-term**	224	222	1	2.4 (1.4, 4.0) *
	**long-term**	355	353	2	2.7 (1.5, 4.7) *
Daily cigarette consumption	**long-term**	131	131	1	20.7 (19.8, 21.6)*
Saliva cotinine (μg/L)	**short-term**	40	40	2	-0.2 (-0.3, -0.2) *
**2. Real acupuncture versus sham acupuncture**					
Abstinence rate	short-term	96	89	3	1.8 (0.9, 3.3)
	mid-term	36	33	1	1.4 (0.4, 4.5)
	long-term	37	27	2	0.8 (0.2, 2.9)
Nicotine withdrawal symptoms (NWS)	short-term	81	83	2	-0.7 (-1.7, 0.3)
Beck Depression Inventory (BDI)	short-term	22	24	1	0.9 (-1.6, 3.4)
Beck Anxiety Inventory (BAI)	short-term	22	24	1	-0.4 (-2.3, 1.5)
Craving for cigarette	short-term	10	14	1	1.6 (0.9, 2.9)
Daily cigarette consumption	**short-term**	11	12	1	-5.1 (-7.0, -3.2) *
	**long-term**	22	11	1	-3.2 (-5.1, -1.4) *
Exhaled CO level (ppm)	**short-term**	15	14	1	-0.6 (-0.9, -0.3) *
Serum cotinine (μg/L)	**short-term**	26	18	1	-86.0 (-107.8, -64.2) *
	**long-term**	22	16	1	-80.0 (-100.0, -60.0) *
**3. Acupuncture versus nicotine replacement therapy (NRT)**					
Abstinence rate	short-term	224	205	1	1.04 (1.0, 1.2)
	long-term	224	205	1	1.05 (1.0, 1.1)
Nicotine withdrawal symptoms (NWS)	short-term	30	30	1	-2.3 (-20.3, 15.7)
Fagerström test for nicotine dependence (FTND)	short-term	24	23	1	0.5 (-0.3, 1.3)
	mid-term	24	23	1	0.1 (-0.7, 0.9)
Heaviness of smoking index (HSI)	short-term	24	23	1	0.3 (-0.3, 0.8)
	mid-term	24	23	1	0.1 (-0.5, 0.7)
Exhaled CO level (ppm)	short-term	24	23	1	-0.5 (-2.6, 1.7)
	mid-term	24	23	1	0.4 (-2.1, 2.8)
Urine cotinine (μg/L)	short-term	24	23	1	-0.2 (-1.1, 0.8)
	mid-term	24	23	1	-0.3 (-1.1, 0.4)
**4. Acupuncture versus acupuncture combined with counselling or educational smoking cessation program or moxibustion**					
Abstinence rate	**short-term**	236	208	3	0.8 (0.6, 0.9) *
	mid-term	169	162	2	0.8 (0.6, 1.1)
	**long-term**	166	165	2	0.6 (0.4, 0.8) *
**5. Acupuncture versus wrist-ankle acupuncture**					
Abstinence rate	long-term	30	30	1	0.6 (0.4, 0.9)
**6. Acupuncture combined with auricular acupressure versus sham acupuncture combined with sham auricular acupressure**					
Abstinence rate	**short-term**	100	97	3	2.5 (1.4, 4.3) *
	**mid-term**	70	67	2	3.6 (1.4, 9.5) *
Fagerström test for nicotine dependence (FTND)	**short-term**	30	30	1	-3.9 (-5.1, -2.7) *
	**mid-term**	30	30	1	-2.8 (-4.1, -1.5) *
Self-evaluated scale for tobacco dependence (SSTD)	**short-term**	30	30	1	-9.5 (-12.4, -6.6) *
	**mid-term**	30	30	1	-4.8 (-7.9, -1.7) *
Daily cigarette consumption	**short-term**	60	60	2	-6.9 (-9.6, -4.3) *
	**mid-term**	30	30	1	-8.9 (-12.6, -5.3) *
Exhaled CO level (ppm)	short-term	30	29	1	-3.2 (-7.5, 1.0)
Serum cotinine (μg/L)	short-term	30	29	1	-21.7 (-105.1, 61.6)
**7. Acupuncture combined with auricular acupressure versus acupuncture or auricular acupressure as a monotherapy**					
Abstinence rate	**short-term**	76	74	3	1.5 (1.0, 2.3) *
Fagerström test for nicotine dependence (FTND)	**short-term**	24	24	1	-2.1 (-3.1, -1.2) *
Self-evaluated scale for tobacco dependence (SSTD)	**short-term**	24	24	1	-6.3 (-9.1, -3.4) *
Daily cigarette consumption	**short-term**	24	24	1	-5.0 (-9.7, -0.4) *
**8. Acupuncture combined with auricular acupressure versus nicotine replacement therapy (NRT)**					
Abstinence rate	short-term	56	60	1	0.6 (0.3, 1.4)
	mid-term	30	32	1	1.3 (0.4, 3.78)
Nicotine withdrawal symptoms (NWS)	short-term	30	32	1	-0.5 (-2.9, 1.9)
	mid-term	30	32	1	-0.5 (-2.4, 1.4)
Fagerström test for nicotine dependence (FTND)	short-term	60	62	2	0.1 (-0.5, 0.7)
Heaviness of smoking index (HSI)	short-term	60	62	2	-0.1 (-0.4, 0.4)
Brief Questionnaire of Smoking Urges (QSU-Brief)	short-term	30	32	1	-1.0 (-4.5, 2.5)
	mid-term	30	32	1	-0.9 (-4.4, 2.5)
**9. Acupuncture combined with counseling/gum versus sham acupuncture combined with counselling/gum**					
Abstinence rate	short-term	425	376	2	1.0 (0.9, 1.2)
	mid-term	146	145	1	0.99 (0.7, 1.3)
	long-term	417	355	2	0.99 (0.7, 1.3)

Short-term, for assessment up to 4 weeks; Mid-term, for an assessment duration up to 6 months; Long-term, for an assessment duration longer than 6 months. *p<0.05 and the difference was statistically significant. RR: risk ratio, used for dichotomous data-abstinence rate, MD: mean difference, used for continuous data-all outcomes except abstinence rate, CI: confidence interval, for RR the exclusion of the value 1 implies statistical significance whereas for MD the exclusion of the value 0 implies statistical significance.

#### Real acupuncture versus sham acupuncture

##### Abstinence rate

Real acupuncture showed no difference to sham acupuncture on smoking cessation in the short-term (3 trials, RR=1.78, 95% CI: 0.96, 3.27), mid-term (1 trial, RR=1.38, 95% CI: 0.43, 4.45) and long-term (2 trials, RR=0.80, 95% CI: 0.23, 2.85) follow-up.

##### Nicotine withdrawal symptoms

Two trials reported outcomes of NWS at short-term follow-up. Meta-analysis showed that real acupuncture was not superior to sham acupuncture (2 trials, MD= -0.72, 95% CI: -1.72, 0.28).

##### Beck Depression Inventory

There was only one trial reporting BDI at short-term follow-up; real acupuncture was not superior sham acupuncture (1 trial, MD=0.90, 95% CI: -1.56, 3.36).

##### Beck Anxiety Inventory

In one study, real acupuncture showed no effect compared with sham acupuncture at short-term follow-up (1 trial, MD= -0.40, 95% CI: -2.28, 1.48).

##### Exhaled CO level (ppm)

We found one study reporting exhaled CO level, and real acupuncture was more effective in short-term than sham acupuncture (1 trial, MD= -0.60, 95% CI: -0.93, -0.27).

##### Serum cotinine (μg/L)

One study reported outcomes of serum cotinine. Real acupuncture demonstrated a better effect than sham acupuncture at short-term follow-up (1 trial, MD= -86.00, 95% CI: -107.79, -64.21) and long-term point (1 trial, MD= -80.00, 95% CI: -100.03, -59.97).

##### Daily cigarette consumption

Two trials reported outcomes of daily cigarette consumption, one of these in the short-term and another in the long-term. Real acupuncture showed a better effect than sham acupuncture at short-term (1 trial, MD= -5.10, 95% CI: -6.99, -3.21) and long-term follow-up (1 trial, MD= -3.20, 95% CI: -5.05, -1.35).

##### Craving for cigarette

We found one study reporting craving for cigarette, and the comparison between real acupuncture and sham acupuncture was not statistically different (1 trial, MD=1.60, 95% CI: 0.87, 2.94) at short-term follow-up.

#### Acupuncture versus nicotine replacement therapy

##### Abstinence rat

There was only one study reporting smoking cessation. Acupuncture was not superior to NRT at short-term (1 trial, RR=1.04, 95% CI: 0.95, 1.15) and long-term (1 trial, RR=1.05, 95% CI: 0.98, 1.11) follow-up.

##### Nicotine withdrawal symptoms

Acupuncture was not superior to NRT at short-term follow-up (1 trial, MD= -2.30, 95% CI: -20.29, 15.69).

##### Fagerström test for nicotine dependence

One study reported outcomes of FTND. Acupuncture was not superior to NRT at the short-term (1 trial, MD=0.48, 95% CI: -0.31, 1.27) and long-term point (1 trial, MD=0.10, 95% CI: -0.71, 0.91) follow-up.

##### Exhaled CO level (ppm)

Acupuncture was not superior to NRT at short-term (1 trial, MD= -0.49, 95% CI: -2.64, 1.66) and mid-term (1 trial, MD=0.36, 95% CI: -2.11, 2.83) follow-up.

##### Urine cotinine (μg/L)

Acupuncture was not superior to NRT at short-term (1 trial, MD= -0.18, 95% CI: -1.14, 0.78) and mid-term (1 trial, MD= -0.34, 95% CI: -1.11, 0.43) follow-up.

##### Heaviness of smoking index

Acupuncture was not superior to NRT at short-term (1 trial, MD=0.28, 95% CI: -0.28, 0.84) and mid-term (1 trial, MD=0.11, 95% CI: -0.50, 0.72) follow-up.

#### Acupuncture versus no intervention/waiting list

##### Abstinence rate

One trial reported outcome of smoking cessation at short-term follow-up. Acupuncture was superior to no intervention/waiting list control (1 trial, RR=2.37, 95% CI: 1.41, 3.97). Two trials reported outcome of smoking cessation at long-term follow-up, pooled results comparing acupuncture to no intervention/ waiting list demonstrated superiority of acupuncture (2 trials, RR=2.66, 95% CI: 1.50, 4.70).

##### Saliva cotinine (μg/L)

Meta-analysis indicated that acupuncture was more effective than no intervention/waiting list control related to saliva cotinine (2 trials, MD= -0.21, 95% CI: -0.26, -0.17) in short-term.

##### Reduction of daily cigarette consumption

In one study, acupuncture showed a greater reduction in daily cigarette consumption compared to no intervention/waiting list control for reduction outcome of daily cigarette consumption at long-term follow-up (1 trial, MD=20.70, 95% CI: 19.83, 21.57).

#### Acupuncture combined with auricular acupressure versus acupuncture or auricular acupressure as a monotherapy

##### Abstinence rate

The pooled analysis suggested that acupuncture combined with auricular acupressure increased the short-term cessation of smokers compared to acupuncture or auricular acupressure as monotherapy (3 trials, RR=1.52, 95% CI: 1.03, 2.25).

##### Fagerström test for nicotine dependence

We found one study reporting FTND, and the comparison between acupuncture combined with auricular acupressure and auricular acupressure as a monotherapy with a higher FTND score reduction in the combined group at short-term follow-up (1 trial, MD= -2.13, 95% CI: -3.11, -1.15).

##### Self-evaluated scale for tobacco dependence

One study reported outcomes on a self-evaluated scale for tobacco dependence. Acupuncture combined with auricular acupressure demonstrated a better effect than auricular acupressure as a monotherapy at short-term follow-up (1 trial, MD= -6.29, 95% CI: -9.14, -3.44).

##### Daily cigarette consumption

One study reported outcomes of daily cigarette consumption. Acupuncture in combination with auricular acupressure had a better effect than auricular acupressure at short-term follow-up (1 trial, MD= -5.04, 95% CI: -9.73, -0.35).

#### Acupuncture combined with auricular acupressure versus sham acupuncture combined with sham auricular acupressure

##### Abstinence rate

The pooled analysis from three studies suggested an increase in quit rates applying acupuncture plus auricular acupressure compared to sham acupuncture plus sham auricular acupressure (3 trials, RR=2.50, 95% CI: 1.44, 4.33) at short term follow-up. Results from two studies showed an increase in cessation rates using acupuncture combined with auricular acupressure compared to sham acupuncture plus sham auricular acupressure (2 trials, RR=3.61, 95% CI: 1.37, 9.48) at mid-term follow-up.

##### Fagerström test for nicotine dependence

Acupuncture combined with auricular acupressure was able to reduce the score of FTND compared to sham acupuncture plus sham auricular acupressure at short-term (1 trial, MD= -3.87, 95% CI: -5.07, -2.67) and mid-term (1 trial, MD= -2.77, 95% CI: -4.09, -1.45) follow-up.

##### Self-evaluated scale for tobacco dependence

Acupuncture combined with auricular acupressure was superior in decreasing the score of a self-evaluated scale for tobacco dependence to sham acupuncture plus sham auricular acupressure at short-term (1 trial, MD= -9.53, 95% CI: -12.42, -6.64) and mid-term (1 trial, MD= -4.79, 95% CI: -7.85, -1.73) follow-up.

##### Exhaled CO level (ppm)

Acupuncture combined with auricular acupressure relative to sham acupuncture plus sham auricular acupressure was not significantly associated with a decrease in exhaled CO level (1 trial, MD= -3.24, 95% CI: -7.48, 1.00) at short-term follow-up.

##### Serum cotinine (μg/L)

In one trial, acupuncture combined with auricular acupressure was not superior to sham acupuncture plus sham auricular acupressure reducing serum cotinine at short-term (1 trial, MD= -21.74, 95% CI: -105.12, 61.64) follow-up.

##### Daily cigarette consumption

We found two studies reporting daily cigarette consumption. Acupuncture combined with auricular acupressure compared to sham acupuncture plus sham auricular acupressure significantly decreased daily cigarette consumption at short-term (2 trials, MD= -6.96, 95% CI: -9.64, -4.28) and mid-term (1 trial, MD= -8.96, 95% CI: -12.58, -5.34) follow-up.

#### Acupuncture combined with auricular acupressure versus nicotine replacement therapy

##### Abstinence rate

Acupuncture combined with auricular acupressure did not have superior quit rates compared to NRT at short-term (2 trials, RR=0.61, 95% CI: 0.28, 1.36) and mid-term (1 trial, RR=1.28, 95% CI: 0.44, 3.76) follow-up.

##### Nicotine withdrawal symptoms

The difference of NWS score between acupuncture combined with auricular acupressure and NRT was not statistically significant at short-term (1 trial, MD= -0.50, 95% CI: -2.85, 1.85) and mid-term (1 trial, MD= -0.50, 95% CI: -2.36, 1.36) follow-up.

##### Fagerström test for nicotine dependence

Meta-analysis from two studies suggested no statistically significant difference between acupuncture combined with acupressure and NRT in decreasing the FTND score at short-term (2 trials, MD=0.11, 95% CI: -0.49, 0.71) follow-up.

##### Heaviness of Smoking Index

Meta-analysis showed no statistically significant difference between acupuncture combined with acupressure and NRT in the change in HSI at short-term (2 trials, MD= -0.04, 95% CI: -0.42, 0.35) follow-up.

##### Brief Questionnaire of Smoking Urges

Acupuncture combined with auricular acupressure was not superior to NRT in reducing the QSU-Brief score at short-term (1 trial, MD= -1.03, 95% CI: -4.52, 2.46) and mid-term (1 trial, MD= -0.93, 95% CI: -4.35, 2.49) follow-up.

#### Acupuncture versus acupuncture combined with counselling or educational smoking cessation program or moxibustion

Meta-analysis demonstrated that acupuncture was less effective in reducing cessation rates compared to acupuncture plus counselling/educational smoking cessation program/moxibustion at short-term (3 trials, RR=0.75, 95% CI: 0.63, 0.91) and long-term (2 trials, RR=0.77, 95% CI: 0.56, 1.05) follow-up. However, there was no significant difference between the treatment groups at mid-term (2 trials, RR=0.56, 95% CI: 0.38, 0.82) follow-up.

#### Acupuncture versus wrist-ankle acupuncture

Acupuncture was less effective than wrist-ankle acupuncture in reducing cessation rates at short-term (1 trial, RR=0.64, 95% CI: 0.41, 0.99) follow-up.

#### Acupuncture combined with counselling/gum versus sham acupuncture combined with counselling/gum

Meta-analysis demonstrated that acupuncture plus counselling/gum was not superior to sham acupuncture plus counselling/gum in reducing cessation at short-term (2 trials, RR=1.03, 95% CI: 0.87, 1.21), mid-term (1 trials, RR=0.99, 95% CI: 0.74, 1.34), and long-term (2 trials, RR=1.06, 95% CI: 0.78, 1.44) follow-up.

### Funnel plot analysis

Since there were less than five studies in all metaanalyses, we did not use funnel plots to assess publication bias. It is possible that the results of this review are at risk of publication bias, because we identified a trial registered in Chinese Clinical Trial Registry (ChiCTR-TRC-13003544) that had not been published since completion.

### Trial sequential analysis

TSA^68^ was conducted to estimate the required sample size for a systematic review based on primary outcome of different comparisons and to detect the robustness of the result. The estimate of required information size (RIS) was based on type I error (α=5%), type II error (β=20%), relative risk reduction (RRR=20.0%) and cessation rate in control group. The TSA results are given in Appendix 2.

TSA illustrated that the cumulative Z-curve (blue curve) of acupuncture versus acupuncture combined with counselling/educational smoking cessation program/moxibustion in the long-term going across the traditional boundary of 5% significance (green curve) and crossing the monitoring boundaries (red inward sloping curves). This means that although the cumulative sample size did not meet expectations, no more trials were needed and a positive conclusion was reached in advance. By adjusting the random error, we can conclude that acupuncture combined with counselling/educational smoking cessation program/ moxibustion results in a higher rate of long-term abstinence rate than acupuncture alone. However, the cumulative Z-curve (blue curve) of other comparisons or follow-up terms whether or not across the traditional boundary of 5% significance (green curves) did not cross the monitoring boundaries (red inward sloping curves) and RIS boundary (red vertical line), which was needed to obtain firm evidence controlling for the risk of random error. TSA of these included trials suggests that high quality RCTs are required to confirm possible intervention effects.

### Adverse events

Of the included 24 trials, there were 13 trials^45-47,49-53,58-61,67^ (13/24; 54.17%) that did not give any information on adverse events. And no serious adverse events occurred in the remaining 11 trials (11/24; 45.83%). Four trials^54,57,62,64^ (4/24; 16.67%) reported that no adverse events occurred. Another four trials^48,55,56,63^ (4/24; 16.67%) reported infrequent minor bleeding or bruising upon needle removal. Non-serious adverse effects including fainting, pain, weeping, anorexia, or headache were observed in association with electroacupuncture in one trial^65^ (1/24; 4.17%). One trial^66^ (1/24; 4.17%) reported side effects during the period of auricular acupuncture therapy including tenderness sensation, feeling, dizziness, minor bleeding, and nausea sensation. In one trial^44^ (1/24; 4.17%) with acupuncture, a participant experienced dizziness as he had not eaten breakfast.

## DISCUSSION

### Main findings

The goal of this systematic review was to evaluate the evidence regarding the effect of transcutaneous acupuncture on smoking cessation. We evaluated 24 RCTs with 3984 participants published from 1982 to February 2017. These studies varied considerably with regard to relevant participant characteristics, interventions, and outcome measures. The quality of evidence was low due to the overall high or unclear risk of bias. We classified comparisons based on the types of experimental and control interventions, which possibly explains why no significant statistical heterogeneity existed, except for one pooled analysis comparing acupuncture in combination with auricular acupressure to sham acupuncture plus sham auricular acupressure on the outcome of daily cigarette consumption (heterogeneity of 95%).

From the results of meta-analysis and TSA, the conclusion can be drawn that acupuncture plus counselling/education/moxibustion is more effective for abstinence rate at long-term than acupuncture alone. Apart from this, on the basis of the available evidence, the TSA indicates that it is not possible to draw any firm conclusions with regard to the other comparisons. However, grouping studies by the nature of the comparisons and intervention types yielded the following trends: 1) acupuncture may be more effective than no intervention/waiting list at short- and long-term follow-up; 2) real acupuncture does not appear to be superior to sham acupuncture in every stage of follow-up; 3) acupuncture shows a similar effect to NRT on smoking cessation in this review for whatever periods and indicators; 4) secondary outcomes are consistent with the primary outcome, sometimes secondary outcomes vary more sensitively than the primary outcome; and 5) only one trial^48^ with 60 participants explored the cost-effectiveness of the interventions between two groups. This study demonstrated that acupuncture plus auricular acupressure with beads was more economical than nicotine paste.

Results between different comparisons, follow-up periods and outcome measures were not always consistent. With regard to sham intervention, most outcome measures showed no significant differences between real acupuncture and sham acupuncture, whereas acupuncture in combination with auricular acupressure was superior to sham acupuncture in combination with sham auricular acupressure This may point to a possible synergistic action between acupuncture and auricular acupuncture. Involving follow-up periods, acupuncture plus counselling/ educational smoking cessation program/moxibustion was more effective than acupuncture on cessation rate for the short-term and long-term, but equal for the mid-term. Referring to outcome measures, in the comparison between acupuncture and sham acupuncture, some outcome indicators showed no significant differences including cessation rate, NWS, BDI, BAI, and craving for cigarette, while others showed significant differences including exhaled CO level, serum cotinine, and daily cigarette consumption. This inconsistency may illustrate the complex and changing process of smoking cessation and interventions, as well as the relatively exploratory nature of these secondary outcome measures.

### Limitations of the systematic review

There were several limitations in this systematic review. Firstly, the methodological quality of the evidence was generally poor as the lack of sufficient information about the random sequence generation, allocation concealment, and blinding in most included studies. Secondly, we only included trials that were published in Chinese or English and this could have excluded potentially important studies. In addition, it is possible that the results of this review are at risk of publication bias. There were no funnel plots in this review because there were less than 10 eligible studies per comparison. Finally, participants who dropped out or lost follow-up due to moving or changing contact information were not counted in the efficacy analysis, which may have affected the results.

### Comparison with other systematic reviews

Several systematic reviews have been performed to evaluate the efficacy of acupoint stimulation for smoking cessation. However, none of them evaluated the efficacy of transdermal acupuncture by needles specifically. This review focused on penetrating acupuncture and we evaluated all outcome measures of included trials for three follow-up periods.

The evidence of this paper shows that secondary outcomes are not always consistent with the primary outcome, sometimes secondary outcomes vary more sensitively than the primary outcome. That was a warning to pay attention not only to abstinence rate but also to the secondary outcome measures, in order to evaluate the efficacy of smoking cessation measures earlier and more fully. Acupuncture appears to be equivalent to NRT for smoking cessation for whatever periods and indicators. These findings have not been reported in previous systematic reviews.

Acupuncture may be more effective than no intervention/waiting list in the short-term and long-term, which is inconsistent with a Cochrane review in 2014^37^. Our findings suggest that real acupuncture appears not to be superior to sham acupuncture in every stage of follow-up. This is consistent with the result of previous systemic reviews^38,39^.

One meta-analysis^69^ indicated that multi-modality treatment, especially acupuncture combined with smoking cessation education or other interventions, can help smokers to quit smoking during treatment and to avoid relapse after treatment. This conclusion is consistent with our results.

### Future research

In order to confirm the effect of acupuncture on smoking cessation, future trials should improve the quality of methodology, enlarge the sample size, extend the follow-up period, and increase secondary outcome indicators especially laboratory indicators. The current evidence supports the notion that further studies should focus on acupuncture combined with other TCM therapies and other currently recommended interventions as part of a complex intervention^70^, which may help smokers to quit or avoid relapse more effectively. Acupuncture may be satisfactory for smoking cessation, if we can give smokers the appropriate therapy package according to the stage of smoking cessation^71^ and the specific factors that promote or hinder quitting.

## CONCLUSIONS

This systematic review suggests that acupuncture may be superior to no intervention/waiting list but as effective as sham acupuncture and NRT. Acupuncture is safe on the basis of this review. However, positive findings should be considered as tentative due to the poor methodological quality of the included studies. More rigorous trials, including those of acupuncture in combination with other interventions are needed. Smoking is a complex phenomenon involving physical, psychological, social and other factors. Nicotine is as addictive as other drugs such as heroine and cocaine. Despite effective smoking cessation treatments, there is still a large effectiveness gap, only about 2% of smokers succeed in giving up smoking in any year^72^. Acupuncture in combination with other TCM therapies or other currently recommended interventions may contribute to reducing the enormous personal and public health burden caused by smoking.

## CONFLICTS OF INTEREST

Authors have completed and submitted the ICMJE Form for Disclosure of Potential Conflicts of Interest and none was reported.
