# Supplementary material for: Acupuncture for smoking cessation: A systematic review and meta-analysis of 24 randomized controlled trials
Source: Tob Induc Dis. 2019 Jun 4;17:48. doi: 10.18332/tid/109195 (PMC6662782; doi:10.18332/tid/109195)
Supplement: Supplementary file 1 [file TID-17-48-s1.pdf]

Appendix 1

Literature retrieval strategy

Indicators	Details
Databases	China National Knowledge Infrastructure (CNKI) (1979-02.2017), Chinese Biomedical Database (SinoMed) (1978-02.2017), Chinese Scientific Journal Database (VIP) (1989-02.2017), Wanfang Database (1990-02.2017), Traditional Chinese Medicine online (TCM online) (1949-02.2017), PubMed (1966-02.2017), and the Cochrane Library (1999-02.2017)
Retrieval time	from inception to February 2017
MeSH term	" Acupuncture" and ''Smoking Cessation" or ''Tobacco Use Disorder" or ''Substance Withdrawal Syndrome"
Key words	''acupuncture'' or ''electro*acupuncture'' or ''auricular acupuncture'' or ''laser needle'' or ''scalp acupuncture'' or ''needle-embedding'' or ''Dai Zhen Gao'' and ''smoking cessation'' or ''tobacco cessation'' or ''quit smoking'' or ''stop smoking'' or ''preventing smoking'' or ''withdrawal symptoms'' or ''withdrawal syndrome'' or ''nicotine dependence''
Language	The language of publications were limited in Chinese and English

© 2019 Wang J.
