# Supplementary material for: Acupuncture for smoking cessation: A systematic review and meta-analysis of 24 randomized controlled trials
Source: Tob Induc Dis. 2019 Jun 4;17:48. doi: 10.18332/tid/109195 (PMC6662782; doi:10.18332/tid/109195)
Supplement: Supplementary file 2 [file TID-17-48-s2.pdf]

Appendix 2 The result of TSA

1. Acupuncture versus no intervention/ waiting list

1.1 short-term

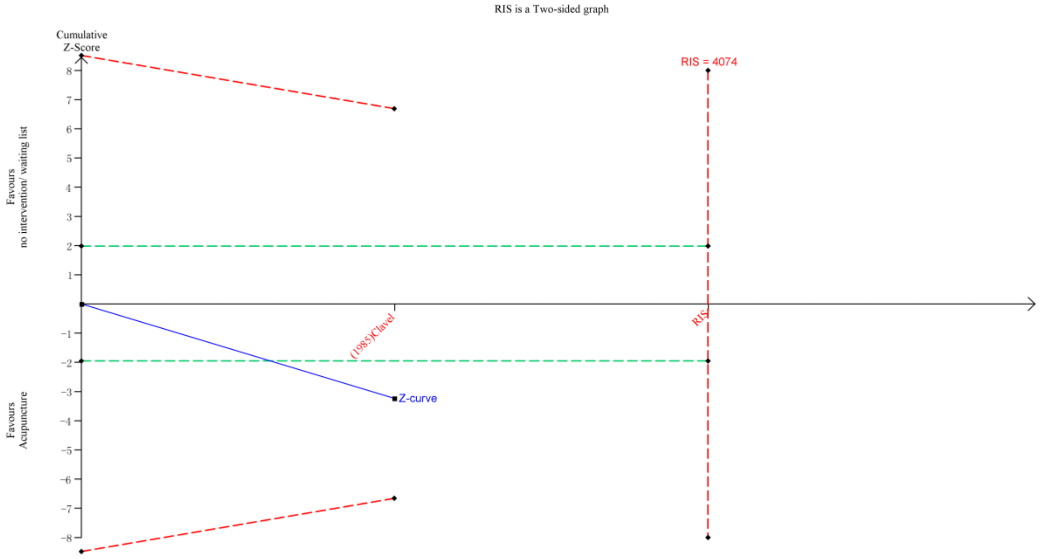

Figure 1. The TSA of short-term abstinence rate of acupuncture versus no intervention/ waiting list

1.2 long-term

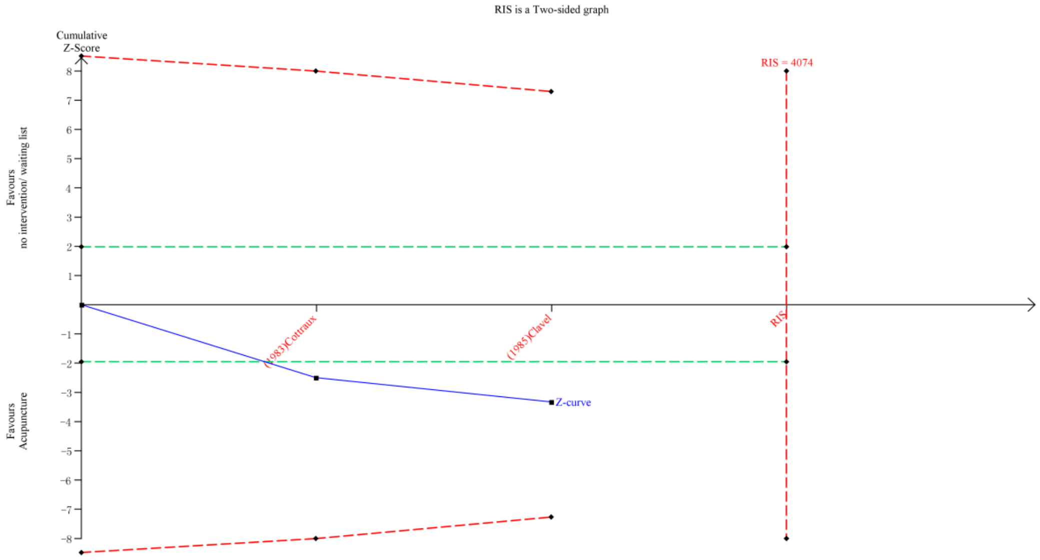

Figure 2. The TSA of long-term abstinence rate of acupuncture versus no intervention/ waiting list

2. Real acupuncture versus sham acupuncture

2.1 short-term

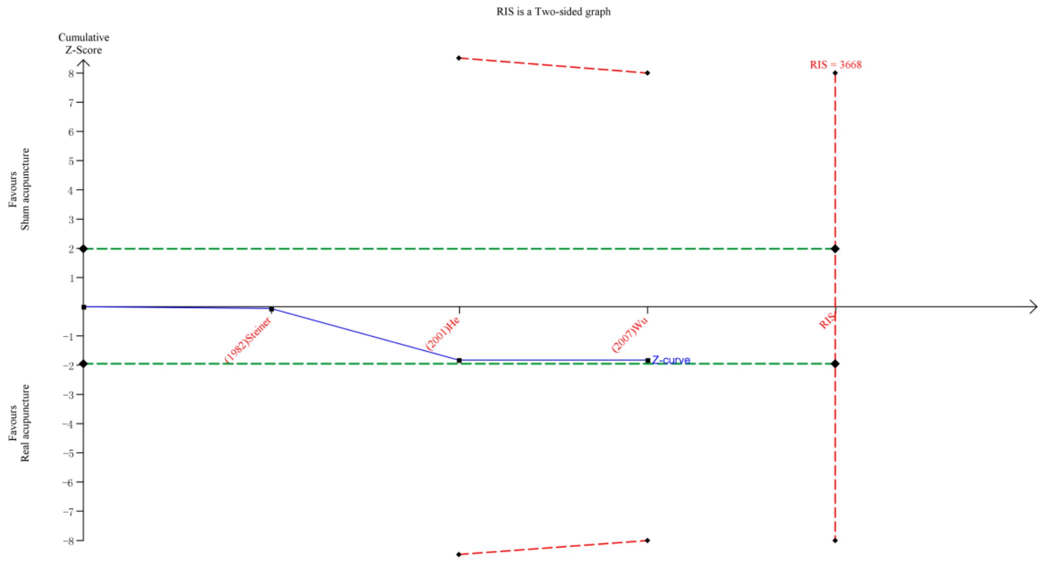

Figure 3. The TSA of short-term abstinence rate of real acupuncture versus sham acupuncture

2.2 mid-term

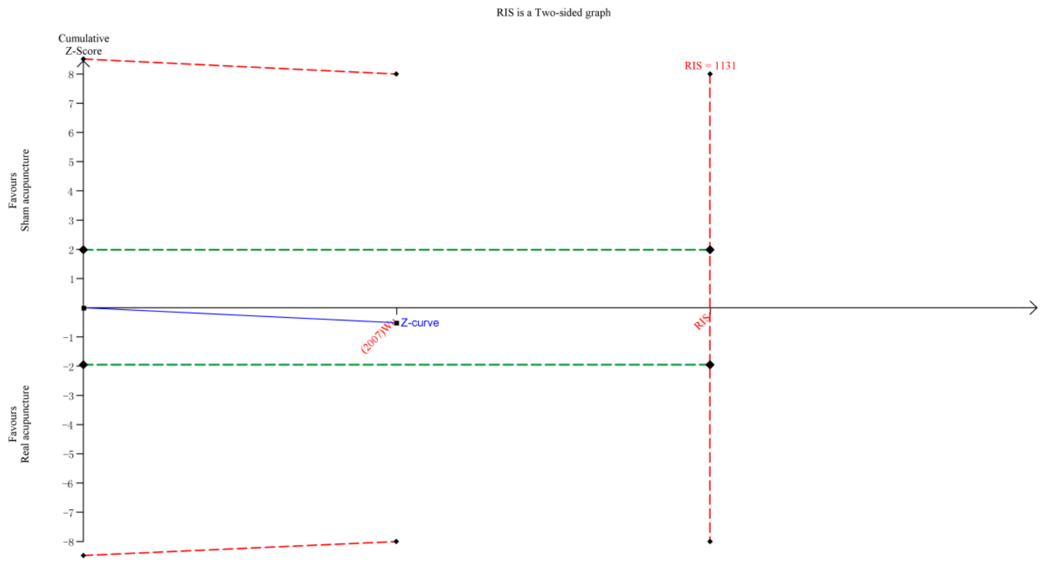

Figure 4. The TSA of mid-term abstinence rate of real acupuncture versus sham acupuncture

2.3 long-term

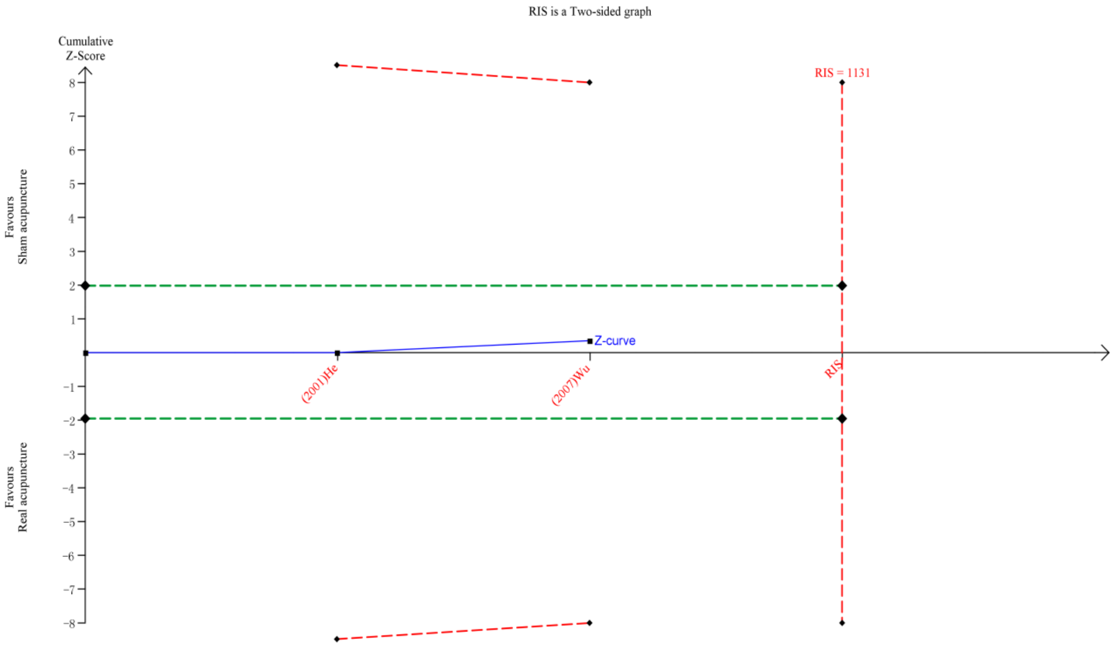

Figure 5. The TSA of long-term abstinence rate of real acupuncture versus sham acupuncture

3. Acupuncture versus nicotine replacement therapy (NRT)

3.1 short-term

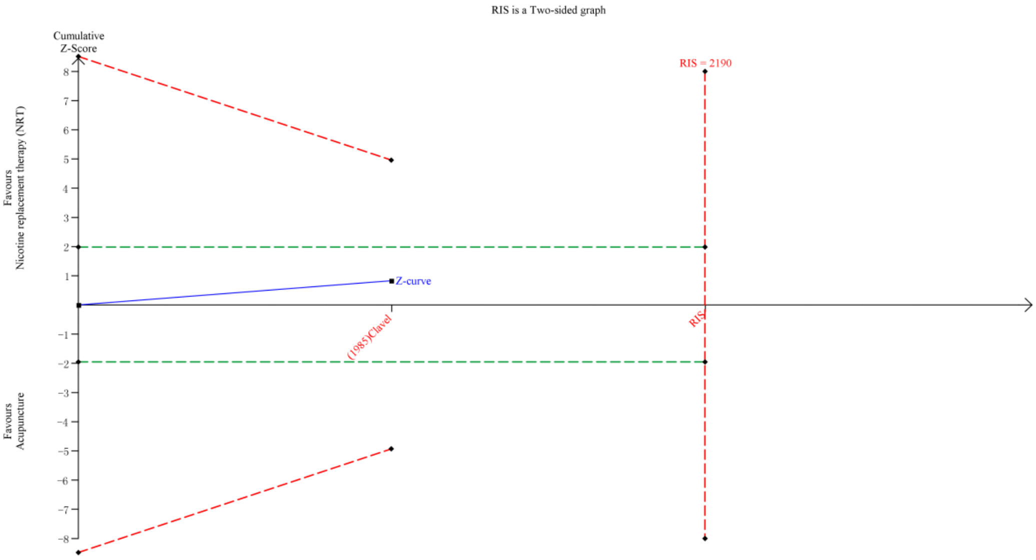

Figure 6. The TSA of short-term abstinence rate of acupuncture versus nicotine replacement therapy (NRT)

3.2 long-term

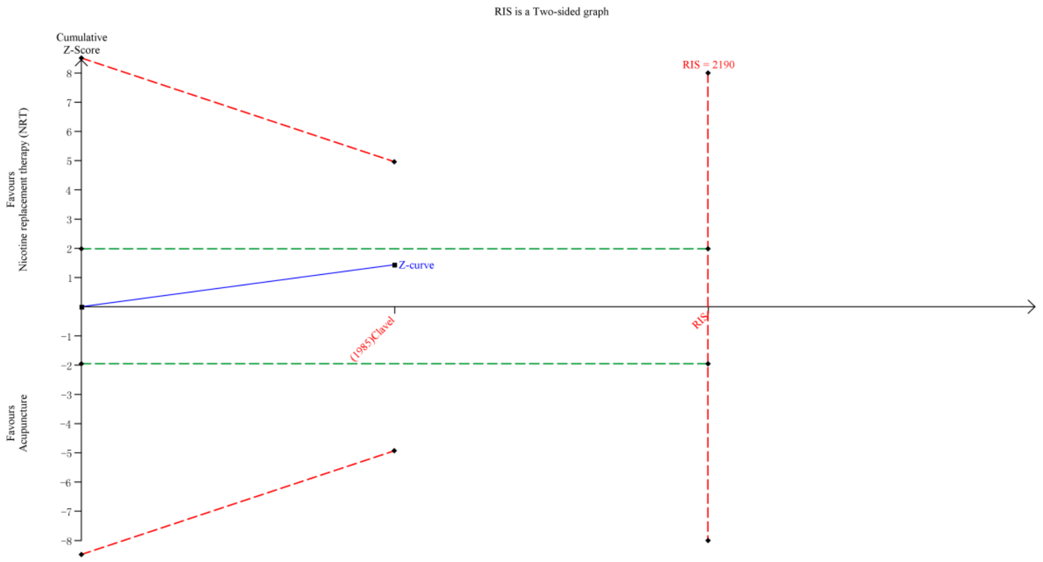

Figure 7. The TSA of long-term abstinence rate of acupuncture versus nicotine replacement therapy (NRT)

4. Acupuncture versus acupuncture combined with counselling or educational smoking cessation program or moxibustion

4.1 short-term

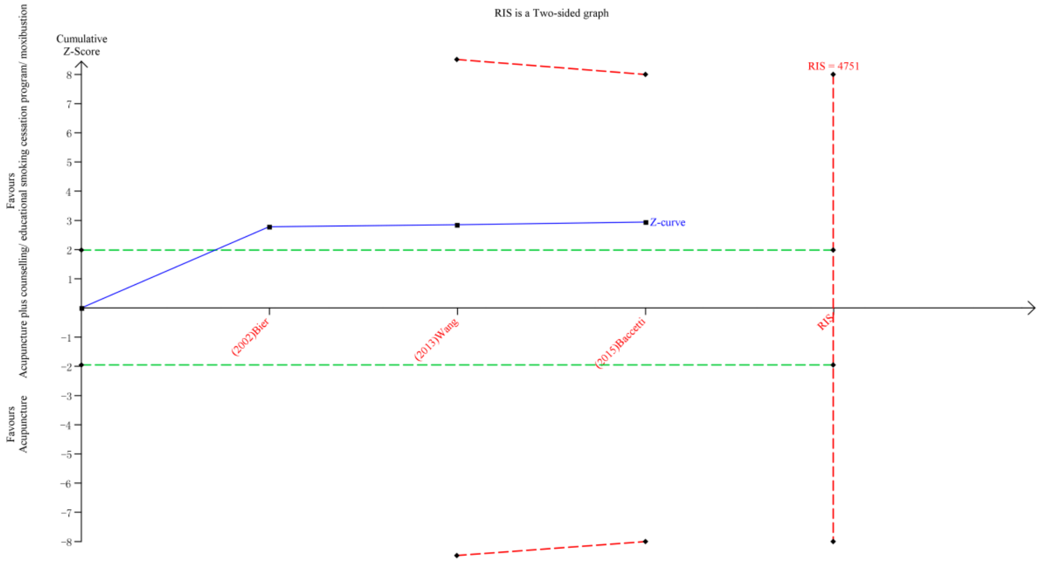

Figure 8. The TSA of short-term abstinence rate of acupuncture versus acupuncture combined with counselling or educational smoking cessation program or moxibustion

4.2 mid-term

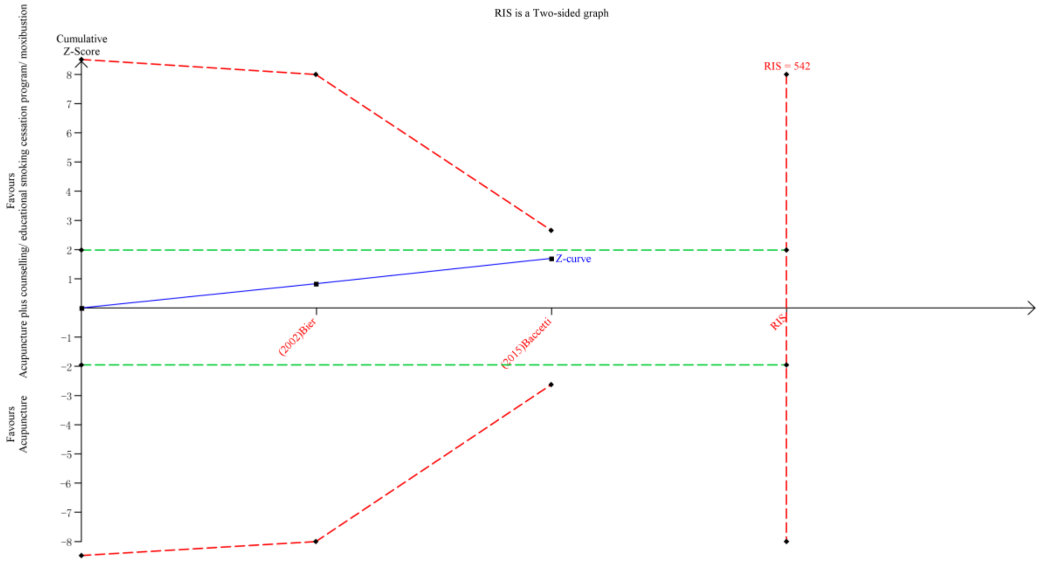

Figure 9. The TSA of mid-term abstinence rate of acupuncture versus acupuncture combined with counselling or educational smoking cessation program or moxibustion

4.3 long-term

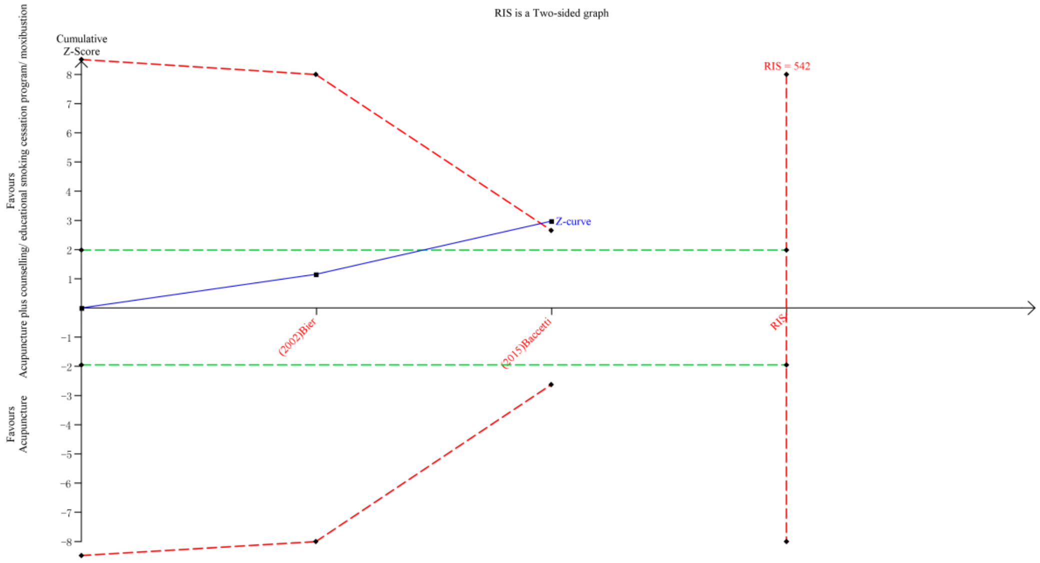

Figure 10. The TSA of long-term abstinence rate of acupuncture versus acupuncture combined with counselling or educational smoking cessation program or moxibustion

5. Acupuncture versus wrist-ankle acupuncture

short-term

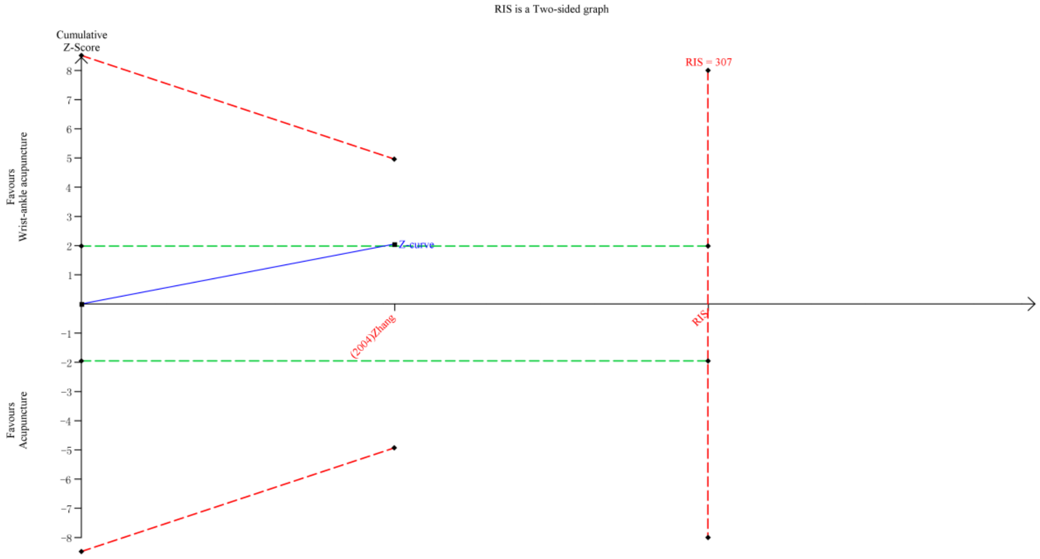

Figure 11. The TSA of short-term abstinence rate of acupuncture versus wrist-ankle acupuncture

6. Acupuncture combined with auricular acupressure versus sham acupuncture combined with sham auricular acupressure

6.1 short-term

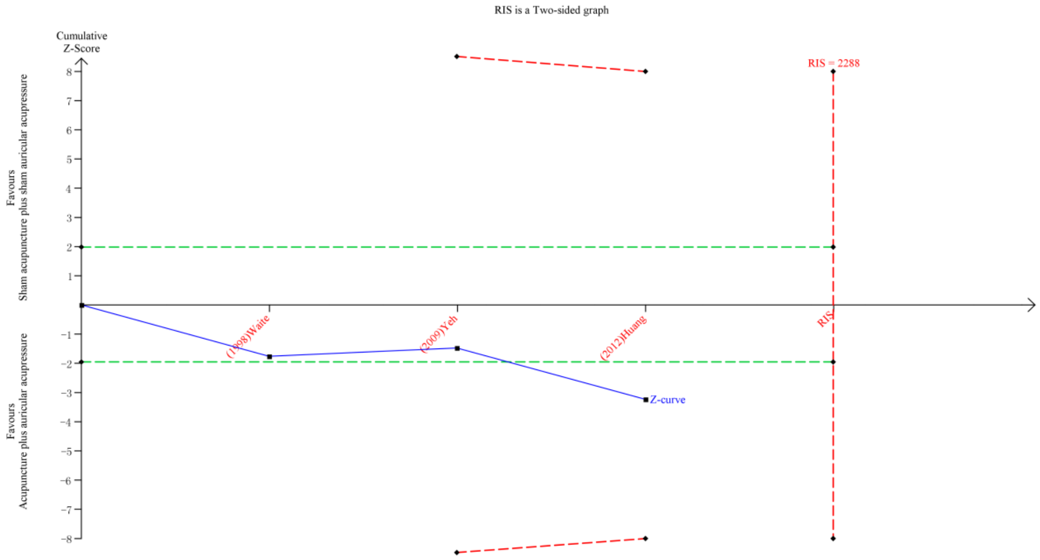

Figure 12. The TSA of short-term abstinence rate of acupuncture combined with auricular acupressure versus sham acupuncture combined with sham auricular acupressure

6.2 mid-term

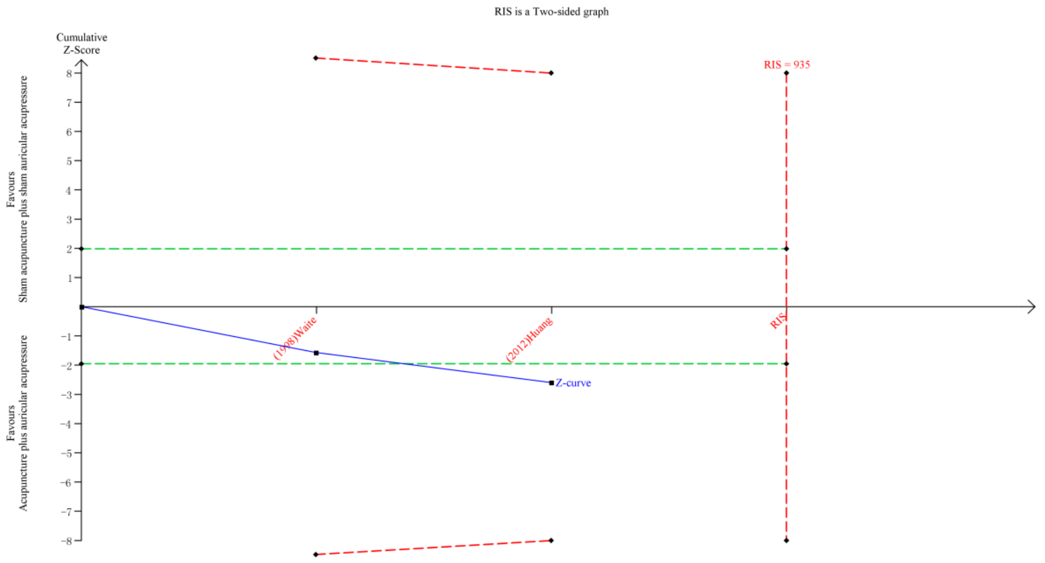

Figure 13. The TSA of mid-term abstinence rate of acupuncture combined with auricular acupressure versus sham acupuncture combined with sham auricular acupressure

7.Acupuncture combined with auricular acupressure versus acupuncture or auricular acupressure as a monotherapy

short-term

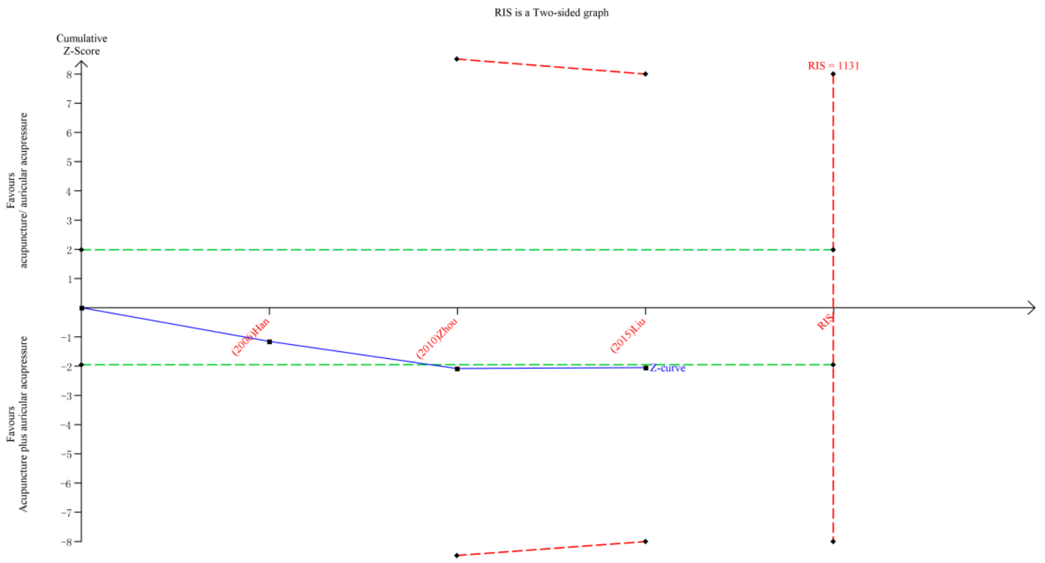

Figure 14. The TSA of short-term abstinence rate of acupuncture combined with auricular acupressure versus acupuncture or auricular acupressure as a monotherapy

8. Acupuncture combined with auricular acupressure versus nicotine replacement therapy (NRT)

8.1 short-term

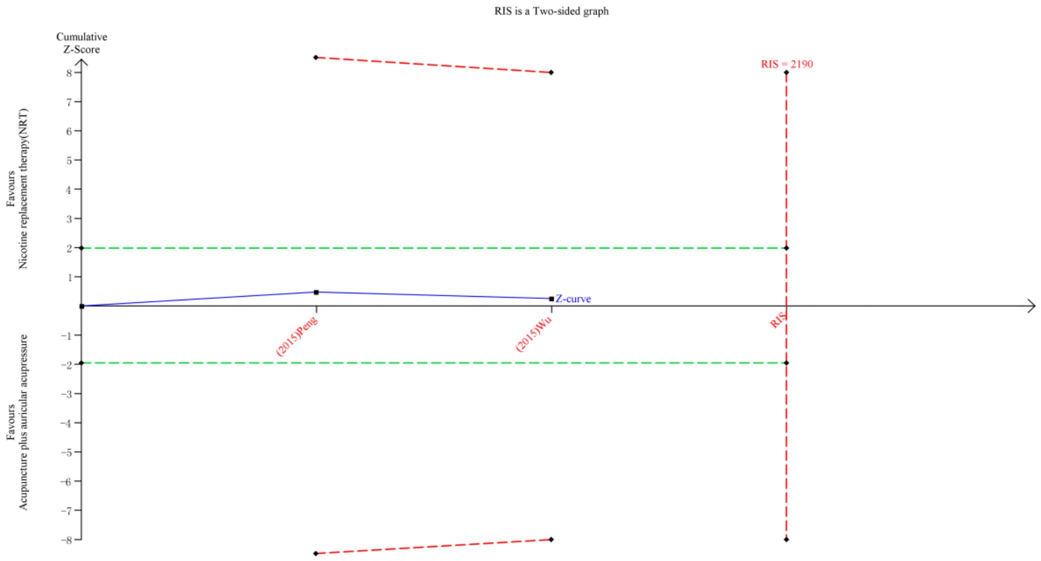

Figure 15. The TSA of short-term abstinence rate of acupuncture combined with auricular acupressure versus nicotine replacement therapy (NRT)

8.2 mid-term

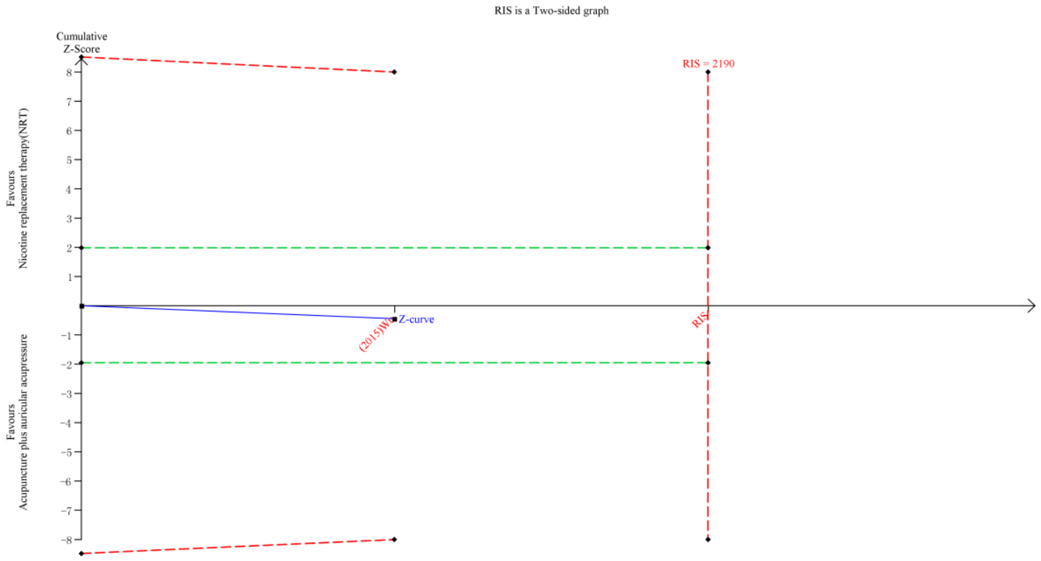

Figure 16. The TSA of mid-term abstinence rate of acupuncture combined with auricular acupressure versus nicotine replacement therapy (NRT)

9. Acupuncture combined with counseling/ gum versus sham acupuncture combined with counselling /gum

9.1 short-term

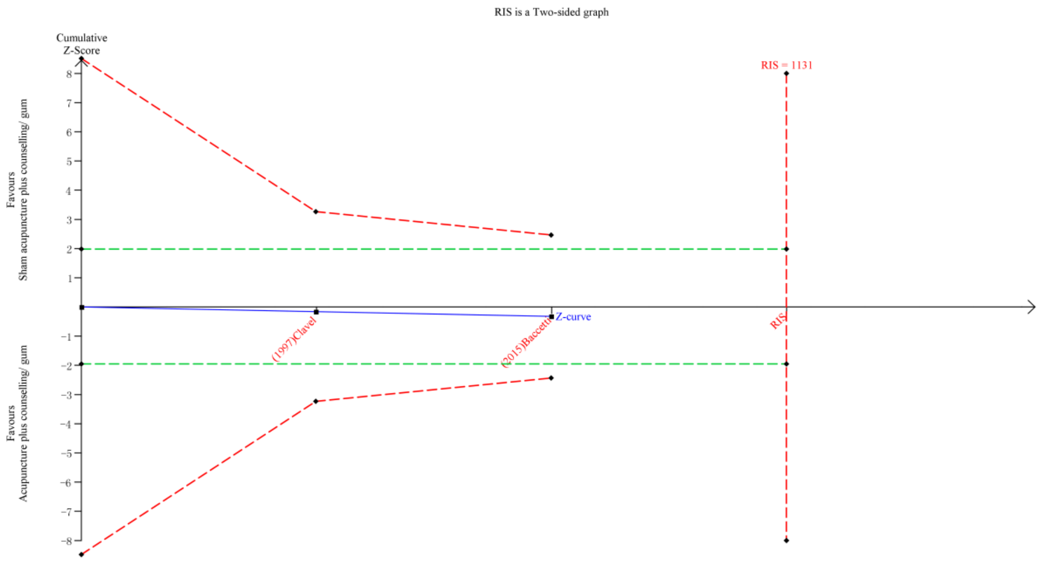

Figure 17. The TSA of short-term abstinence rate of acupuncture combined with counseling/ gum versus sham acupuncture combined with counselling /gum

9.2 mid-term

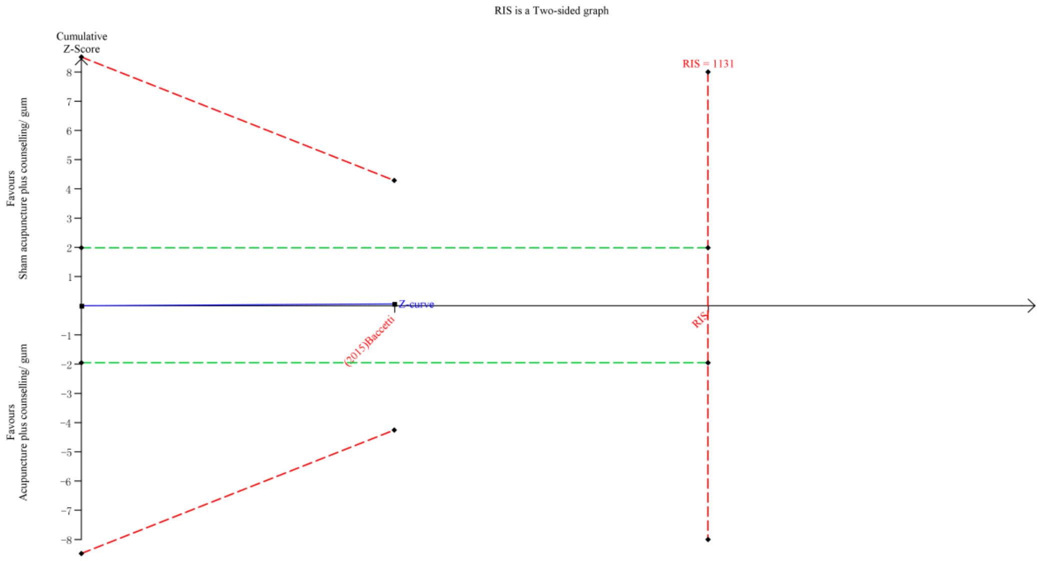

Figure 18. The TSA of mid-term abstinence rate of acupuncture combined with counseling/ gum versus sham acupuncture combined with counselling /gum

9.3 long-term

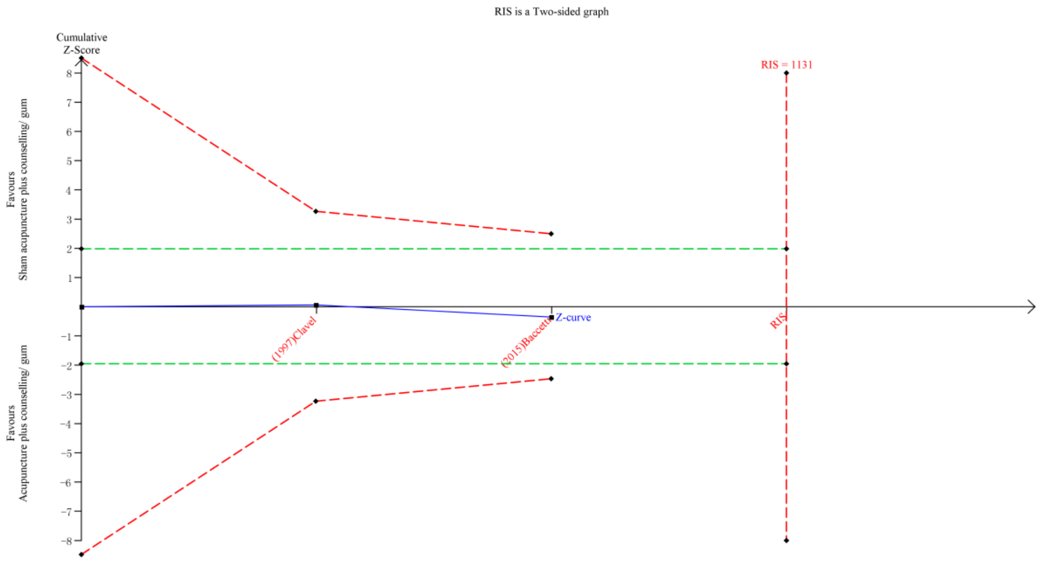

Figure 19. The TSA of long-term abstinence rate of acupuncture combined with counseling/ gum versus sham acupuncture combined with counselling /gum

© 2019 Wang J.
